# Effectiveness of a brief psychoeducational group intervention for relatives on the course of disease in patients after inpatient depression treatment compared with treatment as usual – study protocol of a multisite randomised controlled trial

**DOI:** 10.1186/s12888-015-0633-4

**Published:** 2015-10-23

**Authors:** Fabian Frank, Juliette Wilk, Levente Kriston, Ramona Meister, Shinji Shimodera, Klaus Hesse, Eva-Maria Bitzer, Mathias Berger, Lars P. Hölzel

**Affiliations:** Department of Psychiatry and Psychotherapy, Research Group Psychotherapy and Health Services Research, Medical Center – University of Freiburg, Hauptstraße 5, D-79104 Freiburg, Germany; Department of Public Health and Health Education, University of Education Freiburg, Kunzenweg 21, D-79117 Freiburg, Germany; Department of Medical Psychology, University Medical Center Hamburg-Eppendorf, Martinistraße 52, D-20246 Hamburg, Germany; Department of Neuropsychiatry, Kochi Medical School, Kohasu, Okoh-cho, Nankokushi, Kochi, 783-8505 Japan; Department of Psychiatry and Psychotherapy, University Medical Center Tübingen, Calwerstraße 14, D-72072 Tübingen, Germany

**Keywords:** Depression, Psychoeducation, Family intervention, Relatives, Randomised controlled trial

## Abstract

**Background:**

Relapses and rehospitalisations are common after acute inpatient treatment in depressive disorders. Interventions for stabilising treatment outcomes are urgently needed. Psychoeducational group interventions for relatives were shown to be suitable for improving the course of disease in schizophrenia and bipolar disorders. A small Japanese monocentre randomised controlled trial also showed promising results for depressive disorders. However, the evidence regarding psychoeducation for relatives of patients with depressive disorders is unclear.

**Methods/Design:**

The study is conducted as a two-arm multisite randomised controlled trial to evaluate the incremental effect of a brief psychoeducational group intervention for relatives as a maintenance treatment on the course of disease compared to treatment as usual. Primary outcome is the estimated number of depression-free-days in patients within one year after discharge from inpatient treatment. 180 patients diagnosed with unipolar depressive disorders as well as one key relative per patient will be included during inpatient treatment and randomly allocated to the conditions at discharge. In the intervention group, relatives will participate in a brief psychoeducational group intervention following the patient’s discharge. The intervention consists of four group sessions lasting 90 to 120 min each. Every group session contains informational parts as well as structured training in problem-solving. In both study conditions, patients will receive treatment as usual. Patients as well as relatives will be surveyed by means of questionnaires at discharge and three, six, nine and twelve months after discharge. In addition to the primary outcome, several patient-related and relative-related secondary outcomes will be considered and health economics will be investigated.

**Discussion:**

Our study will provide evidence on the incremental effect of a brief psychoeducational intervention for relatives as a maintenance treatment after inpatient depression treatment. Positive results may have a major impact on health care for depression.

**Trial registration:**

German Clinical Trials Register (DRKS): DRKS00006819; Trial registration date: 2014 Oktober 31; Universal Trial Number (UTN): U1111-1163-5391

## Background

Relapses and rehospitalisations are common in depressive disorders [1–3], although high pre-post effects have been shown for inpatient depression treatment in routine care [4], and a pharmacotherapeutic and/or psychotherapeutic maintenance therapy can reduce the probability of relapses [5]. Interventions for stabilising positive outcomes of depression treatment are therefore urgently needed. For other mental disorders, psychoeducation for relatives has been shown to be suitable for improving patient-relevant outcomes, e.g., relapse, rehospitalisation or employment rate [6–10]. These interventions focus on the information needs of relatives (e.g., [11–13]) as well as on reciprocal interactions between illness-related burden and high expressed emotion in relatives regarding relapses in patients (e.g., [14–16]). For depressive disorders too, the efficacy of a brief psychoeducational group intervention for relatives (PGIR) was initially investigated regarding the relapse rate of patients after discharge from inpatient treatment in a first Japanese randomised controlled trial conducted by Shimazu et al. [17]. Patients of both study conditions were treated fortnightly, with the consultation including an evaluation of depressive symptoms and psychopharmacological and psychotherapeutic treatment. In the intervention group, the relatives took part in a PGIR, which consisted of four group sessions of about 90 to 120 minutes on a fortnightly basis, addressing “epidemiology and causes”, “symptoms”, “treatment and course” and “coping with the patient” as well as group discussions for problem-solving. Relatives participating in the control group did not receive any intervention. The relapse rate in the intervention group was about 8 % within nine months after discharge, in contrast to 50 % in the control group. The study revealed a statistically and clinically significant incremental effect to the psychiatric maintenance treatment. However, in view of differences in health care systems [18] as well as cultural differences, e.g., regarding beliefs about mental illnesses or denotation of symptoms [19, 20], the intervention and the results of the study cannot be directly transferred to Western countries. Moreover, the study was monocentric, with only a small sample size (*N* = 57). As small monocentre trials tend to show higher effect sizes, and results are statistically more uncertain than in multicentre trials with a larger sample size [21, 22], the results are further restricted. The primary endpoint of the study was the time until relapse occurred, which is a clinically relevant criterion. However, the phasic course of depressive disorders is insufficiently reflected by this criterion. Despite these limitations, the results of Shimazu et al. [17] are encouraging, and are in line with results for psychoeducation for relatives in other mental disorders [7–10]. Therefore, the intervention of Shimazu et al. [17] was culturally adapted [23]. As randomised controlled multicentre trials are lacking, and the evidence on the effects of PGIR as a maintenance treatment after inpatient depression treatment is unclear, further research is required to close this evidence gap.

### Objectives

The aim of our study is to evaluate the incremental effect of a short PGIR as a maintenance treatment after inpatient depression treatment in improving the course of illness in patients compared to treatment as usual (TAU). We hypothesise that PGIR is more effective in improving the course of illness than TAU alone. Additionally, we aim to assess secondary outcomes on the patient and relative level as well as health economics on a societal level.

## Methods/Design

### Study design

The effects of a brief PGIR as a maintenance treatment after inpatient depression treatment will be investigated by means of a two-arm parallel-group randomised controlled multicentre trial using five measurements on the patient and on the relative level within one year after discharge of the patient (T_0_ = at discharge; T_1_ = 3; T_2_ = 6; T_3_ = 9; T_4_ = 12 months after discharge). The following eight study centres will be involved in the trial:

1. Department of Psychiatry and Psychotherapy, Medical Centre – University of Freiburg (primary study centre) combined with the Department of Psychosomatic Medicine and Psychotherapy, Medical Centre for Psychiatry Emmendingen;
2. Department of Psychiatry and Psychotherapy, University Medical Centre Tübingen;
3. Department of Psychiatry and Psychotherapy, University Medical Centre Leipzig;
4. Department of General Psychiatry, University Medical Centre Heidelberg;
5. Department of Psychiatry and Psychotherapeutic Medicine, Municipal Hospital Karlsruhe;
6. Department of Psychiatry and Psychotherapy, Vitos Medical Centre Hadamar;
7. Department of Psychiatry, Psychotherapy and Psychosomatics, Vivantes Hospital am Urban Berlin;
8. Department of General Psychiatry and Psychotherapy West, Medical Centre for Psychiatry Weinsberg.

In order to enhance generalisability, we chose different types of hospitals as study centres, including university medical centres, psychiatric wards of general hospitals as well as large psychiatric centres.

### Oversight of research with human participants

The study has been approved by the Ethics Review Committee of the University Medical Centre Freiburg (number: 381/13) and the local Ethics Review Committees responsible for the participating sites (Ethik-Kommission an der Medizinischen Universität Leipzig, number: 346-14-17112014; Ethik-Kommission an der Medizinischen Fakultät der Eberhard-Karls-Universität und am Universitätsklinikum Tübingen, number 544/2014BO2; Ethik-Kommission der Medizinischen Fakultät Heidelberg, number: S-061/2015; Landesärztekammer Baden-Württemberg, number: B-F-2014-093; Ethik-Kommission bei der Landesärztekammer Hessen, number: MC 291/2014). Written informed consent is provided by all study participants prior to data collection, randomisation and intervention. To foster quality assurance and patients’ safety, an advisory board has been implemented, which advises the study protocol, study procedures and realisation of the study. Additionally, trial sites will be visited by a study monitor twice during the recruitment period to ensure compliance with ethical principles and the study protocol, as well as to check data quality and accuracy.

### Inclusion and exclusion criteria

The study population consists of patient-relative tandems. Patients aged 18 years or older treated primarily for a unipolar depressive disorder according to the International Classification of Disease, 10^th^ revision (ICD-10: F32.xx, F33.xx) will be included. Additionally, a key relative (at least 18 years of age), who is named by the patient, has to be willing to participate in the PGIR. Following current recommendations for psychoeducation in relatives, the relative does not have to be a family member, but the patient does have to feel attached to this person [24]. The participating relatives have to live in close proximity to the respective study centre, as participation in the PGIR has to be feasible. To ensure that data can be gathered by questionnaires and that relatives are able to participate in the PGIR, patients and relatives need to be able to read and write in German. Moreover, patients and relatives have to be able to give informed consent. Patients will be excluded if they are diagnosed with comorbid dementia (ICD-10: F0x.x), substance dependence (ICD-10: F1x.2), schizophrenia (ICD-10: F20.x), schizoaffective disorder (ICD-10: F25.x), bipolar disorder (ICD-10: F31.x) or borderline personality disorder (ICD-10: F60.31). To ensure a high external validity of our results, no additional exclusion criteria are formulated.

### Recruitment and eligibility screening

Patient-relative tandems will be recruited at the above-mentioned sites while patients are being treated in inpatient depression treatment. At each study centre, one therapist, who serves as a contact person for the leading study centre, will be responsible for the recruitment. The recruitment procedure is identical at each site according to the following step-by-step routine:

1. By periodically screening new admissions in routine health records of the respective study centre, potentially eligible patients will be identified;
2. Patients identified as potentially eligible in step one will be informed about the study verbally as well as through written study information;
3. If the patient is willing to participate in the trial, the diagnosis will be confirmed using the International Diagnostic Checklist for ICD-10 [25, 26];
4. If the patient is eligible and informed consent has been obtained, eligibility will be screened in the relative named by the patient and informed consent will be obtained from the relative.

To facilitate a consistent implementation across all sites, each procedure is described in detail in a recruitment guideline. For each recruited and randomised patient-relative tandem, an allowance of € 50 will be paid to the recruiting staff. Recruitment will continue until the target population has been achieved. The enrolment period will extend over 12 months.

### Intervention and control group

The intervention follows that of Shimazu et al. [17], but had to be modified in some aspects in order to fit with German conditions. The process of adaptation was based on the results of our own studies [13, 23, 27]. The PGIR will be conducted in a multifamily setting with one key relative per patient and the patient being absent. Groups will consist of three to five relatives. The PGIR consists of four group sessions, which will be conducted on a fortnightly basis. It will begin after discharge of the patient from inpatient depression treatment. Group sessions will be held by one therapist who possesses at least a Master’s degree or equivalent, and will last 90 to 120 min each. The intervention has two main components: Each group session consists of about 45 min of provision of information, while the remaining time is used for training problem-solving skills following the approach of Nezu et al. [28] as well as Hegel et al. [29]. The informational parts cover (1.) symptoms, epidemiology, course and prognosis of depressive disorders; (2.) support services for relatives as well as sources of personal strength and problem areas; (3.) cause of illness and (4.) treatment of depressive disorders. Problem-solving skill training is a brief intervention which has shown high efficacy in several diseases both in patients [30] and in relatives [31]. The PGIR aims to reduce caregiver burden and expressed emotion by teaching relatives how to systematically solve psychosocial problems. To evaluate the incremental effect of the intervention, the PGIR will be compared to treatment as usual (TAU), which in Germany mostly consists of psychotherapeutic and/or pharmacotherapeutic outpatient maintenance treatment provided by psychotherapists, psychiatrists or general practitioners [5]. In the intervention group, the only additional intervention to TAU will be the PGIR. In the control group, there will be no systematic information or psychoeducational intervention offered to relatives. To fit with routine care conditions, neither the inpatient treatment before discharge nor the patient treatment after discharge will be restricted in any way in the intervention group or the control group.

### Postulated mechanism of action

**Fig. 1:**
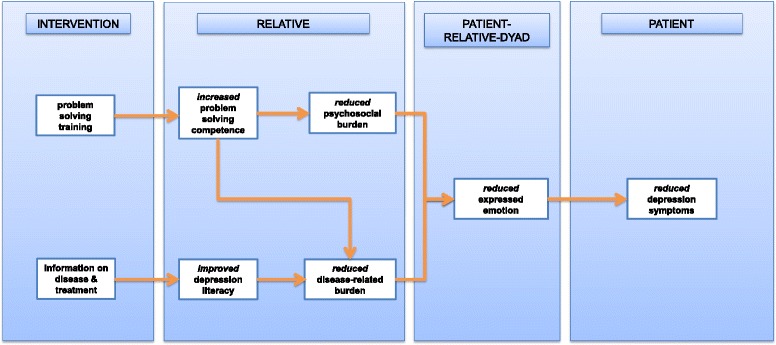
Postulated model of change induced by the PGIR

By introducing information provision and problem-solving skills training as central components of the intervention, the model of change induced by the PGIR is expected to lead to proximal effects on relatives. These effects are thought to influence the patient-relative dyad and might ultimately exert a distal effect by improving the course of illness in patients (c.f. Fig. 1).

First, it is supposed that information provision improves depression literacy in relatives. In this respect, the disease-related burden will be reduced by fostering the understanding and handling of illness-related phenomena [32]. Second, it is assumed that the problem-solving skill training increases the problem-solving competence in relatives. Accordingly, the psychosocial and the disease-related burden will be reduced, as burdensome day-to-day and disease-related problems – e.g., regarding self-care and dealing with the patient – can be more frequently solved. As there are reciprocal interactions between burden on relatives and expressed emotion in relatives [14, 15], these paths are likely to reduce expressed emotion in the patient-relative dyad. Expressed emotion constitutes an indicator of distress in families, taking into account criticism and emotional overinvolvement [16, 33]. It can be distinguished into high expressed emotion and low expressed emotion, with the former having been shown to be a powerful predictor of relapses in patients with depressive disorders after inpatient treatment [16, 34]. Therefore, it is supposed that positive changes on the expressed emotion level might lead to reduced depressive symptoms on the patient level.

### Administration

Patient-relative tandems will be screened for eligibility and recruited by the responsible therapists at the respective study centres during inpatient treatment. Informed consent will be sought from all participating patients and relatives (−T_1_, enrolment, c.f. Table 1). At discharge from inpatient depression treatment, a baseline investigation in patients will be conducted in the respective study centres using questionnaires (T_0_, baseline). At the same time, a baseline investigation in relatives will be centrally provided by the leading study centre using questionnaires sent by post (T_0_, baseline). Subsequent to the baseline investigation, patient-relative tandems will be randomly allocated to the intervention or the control group by the leading study centre (T_A_, allocation). As blinding of patient-relative tandems and therapists is not feasible due to the nature of the intervention, patient-relative tandems as well as therapists will be informed about the allocation by the leading study centre (T_A_, allocation). In the intervention group, the intervention should begin as soon as possible after discharge from inpatient depression treatment (T_Int_, intervention). For each relative participating in the PGIR, the therapist in charge receives an allowance of € 50 per group session. For the follow-up measurements in patients and relatives (T_1_ = 3 months; T_2_ = 6 months; T_3_ = 9 months; T_4_ = 12 months after discharge), postal questionnaires provided by the leading study centre will be used. To ensure a sufficient response rate at each time point, patients as well as relatives will receive an unconditional reimbursement of € 5 sent by post together with each questionnaire. In addition, pre-stamped envelopes will be added for returning the questionnaires, as these measures are associated with a better response rate [35]. If patients and/or relatives do not respond, they will be reminded by post after two weeks and by telephone after four weeks.

**Table 1:** Study schedule and measurements used

	Study period							
	Enrolment	Baseline	Allocation	Intervention	Follow-up			
Time point	-T_1_	T_0_	T_A_	T_Int_	T_1_	T_2_	T_3_	T_4_
Enrolment								
Patients and relatives								
Eligibility screening	x							
Informed consent	x							
Allocation			x					
Intervention								
Psychoeducational group intervention				x				
Adherence and competence of therapists^a/IG^				x				
Self-rating								
Patient-related								
Symptom self-rating (PHQ-9, Core set)		x			x	x	x	x
Rehospitalisations (FIMA, CSSRI-D)					x	x	x	x
Health care and medication consumption (FIMA, CSSRI-D)					x	x	x	x
Employment status^a^		x			x	x	x	x
Days of incapacity to work (DEGS)					x	x	x	x
Functional capability, activity and participation (WHODAS 2.0)		x			x	x	x	x
General state of health (DEGS)		x			x	x	x	x
Perceived expressed emotion (FEF)		x				x		x
Perceived criticism (PCS)		x				x		x
Adverse events^a/IG^						x		x
Clinical characteristics^a^		x						
Demographic information^a^		x						
Relative-related								
Psychosocial burden (SCL-K-9)		x			x	x	x	x
Disease-related burden (IEQ-EU)		x			x	x	x	x
General state of health (DEGS)		x			x	x	x	x
Problem-solving competency (SPSI-R-S)		x				x		x
Implementation of problem-solving^a^						x	x	x
Depression literacy (D-Lit)^a^		x				x		
Control attributions (IPQS-R)		x				x		x
Expressed emotion (FQ)		x				x		x
Health care utilisation (FIMA)		x			x	x	x	x
Employment status^a^		x			x	x	x	x
Acceptance and subjective benefit of the intervention^a/IG^						x		
Adverse Events^a/IG^						x		x
Demographic information^a^		x						

-T_1_, during inpatient treatment; T_0_, at discharge from inpatient treatment; T_A_, allocation to intervention or control group; T_Int_, intervention; Follow-up: T_1_ = 3 months; T_2_ = 6 months; T_3_ = 9 months; T_4_ = 12 months after discharge from inpatient treatment; CSSRI-D, German version of the Client Sociodemographic and Service Receipt Inventory; DEGS, German Health Interview and Examination Survey for Adults; D-Lit, Depression Literacy Test; FEF, Questionnaire on Family Atmosphere; FIMA, Questionnaire for Health-Related Resource Use; FQ, German version of the Family Questionnaire; IEQ-EU, German Version of the Involvement Evaluation Questionnaire; IPQS-R, scales for control attributions of the Illness Perception Questionnaire for Schizophrenia: Relatives’ version; PCS, Perceived Criticism Scale; PHQ-9, 9-item version of the Patient Health Questionnaire; SCL-K-9, 9-item version of the Symptom-Ckecklist-90-R; SPSI-R-S, 25-item version of the Social Problem-Solving Inventory-Revised; WHODAS 2.0, 36-item version of the WHO Disability Assessment Schedule; ^a^self-constructed items; ^IG^only administered in the intervention group

### Primary outcome

The primary outcome is the estimated number of depression-free-days (DFDs) on the patient level. This is a valid and well-established measure which takes into account the course of disease and the change in symptoms over time. Moreover, it is clinically well comprehensible [36, 37]. In a study on the relationship between patient-relative conflicts and long-term depression outcomes, DFDs were proven to be change-sensitive [38]. DFDs will be based on the Patient Health Questionnaire (PHQ-9) [39] measured at five time points (T_0_ – T_4_) and calculated according to Lave et al. [40] and Vannoy et al. [41]. The PHQ-9 shows good psychometric properties [42] and is recommended by the DSM-5 and current guidelines for unipolar depression for measuring symptom severity in depressive disorders [5, 43].

### Secondary outcomes

Secondary outcomes are addressed on the patient and relative level. On a societal level, health economics are considered as a secondary outcome. All measurement instruments employed to measure primary and secondary outcomes as well as all of the measurement time points at which they are applied are shown in Table 1.

#### Patient level

Rehospitalisations within the first year after discharge from inpatient treatment, including time until rehospitalisation, will be considered as a secondary outcome (measurement based on the FIMA [44] and the CSSRI-D [45]; time points T_1_ – T_4_). Furthermore, health care utilisation and medication consumption will be assessed (measurement based on the FIMA [44] and the CSSRI-D [45]; time points T_1_ – T_4_). Additionally, employment status (self-constructed items; time points T_0_ – T_4_) and days of incapacity to work (measurement based on the DEGS [46]; time points T_1_ – T_4_) will be measured as secondary outcomes. Rehospitalisation and incapacity to work are relevant outcomes as they are objective indicators of the course of disease. As these outcomes – as well as health care utilisation and medication consumption – are also associated with costs, they are also important from a health economic point of view. As psychoeducation for relatives was shown to be effective for patients’ employment rate for other severe mental disorders [6], and evidence in depression is so far lacking, this was chosen as a secondary outcome. Depressive disorders can lead to impairment of functional capability, activity and participation [47] and affects the general state of health [48]. Therefore, these are relevant outcomes to judge the effect of the intervention beyond the course of disease (measured by the WHODAS 2.0 [49] and a global item regarding the general state of health according to the DEGS [46]; time points T_0_ – T_4_). As expressed emotion is a powerful predictor of relapses in depressive disorders [16], and the intervention is likely to modify the level of expressed emotion, perceived expressed emotion (measured by the FEF [50, 51]; time points T_0_, T_2_, T_4_) as well as perceived criticism (measured by the PCS [51, 52]; time points T_0_, T_2_, T_4_) are interesting intermediate outcomes to understand how the intervention works. In line with the requirement to report potential harm in patients [53], adverse events following the intervention will be assessed in the intervention group (self-constructed items; time points: T_2_, T_4_).

#### Relative level

Relatives of patients with depressive disorders experience psychosocial burden [54–56]. Therefore, psychological distress (measured by the SCL-K-9 [57]; time points T_0_ – T_4_) and disease-related burden (measured by the IEQ-EU [58, 59]; time points T_0_ – T_4_) were chosen as outcomes to evaluate the effect of the intervention on the relative level. As caregiver burden can affect the health status of relatives [60], the general health status was chosen as an additional outcome to judge the effect of the intervention (measured by a global item regarding the general state of health according to the DEGS [46]; time points: T_0_ – T_4_). As problem-solving competency is considered as a key competency for a healthy family atmosphere [61], this is considered as a secondary outcome (measured by the SPSI-R-S [62, 63]; time points T_0_, T_2_, T_4_). In this regard, the relatives’ implementation of the problem-solving technique conveyed during the intervention is seen as a mediator of the effects (self-constructed items; time points T_2_, T_3_, T_4_). To capture further proximal effects of the intervention, depression literacy will be assessed (measured by the D-Lit [64] and self-constructed items; time points T_0_, T_2_). In addition, control attributions will be examined (measured by scales on control attributions from the IPQS-R [65, 66]; time points T_0_, T_2_, T_4_), as these can be important factors for the development of expressed emotion [65]. Furthermore, expressed emotion will be assessed as an intermediate outcome (measured by the FQ [67]; time points T_0_, T_2_, T_4_). As burden on relatives can also be changed by using various health care services, health care utilisation will be assessed (measurement based on the FIMA [44]; time points T_0_ – T_4_). As caring for a mentally ill person might have an impact on employment activity, employment status will be assessed (self-constructed items; time points T_0_ – T_4_). To judge the intervention from the relatives’ point of view, acceptance and the subjective benefit of the PGIR will be secondary outcomes (self-constructed items; time points T_2_). Following current recommendations [53] and research suggesting potential negative experiences of participants in group settings [68], adverse events will be assessed in the intervention group (self-constructed items; time points T_2_, T_4_).

#### Additional parameters

Data on the following demographic parameters will be collected via self-report questionnaire in patients and relatives: age, sex, mother tongue, education, relationship and shared household with the relative, household size, health insurance status (self-constructed items; time point T_0_). On the patient level, data on clinical characteristics will be gathered regarding: medication, previous depressive episodes and previous inpatient treatment, duration of the current depressive episode, first onset of the depressive disorder and self-rated quality of the current inpatient treatment (self-constructed items; time point T_0_).

#### Health economics

A cost-effectiveness analysis (CEA) will be undertaken from the perspective of the statutory health insurance and the society as a whole. A CEA expresses costs in monetary units and outcomes in non-monetary units. Units used for outcomes will be: (1.) DFDs, (2.) prevented sick leave days, and (3.) prevented rehospitalisations. Costs of TAU and of the intervention will be considered according to German guidelines on health economic evaluation [69]. We will consider both direct medical costs and indirect costs due to loss of productivity. Standardised and validated questionnaires (c.f. Table 1) will be used to obtain data about consumption of health resources [44], which will be monetised following current recommendations [70] to illustrate direct medical costs. The incremental cost-effectiveness ratio of the intervention for DFDs, prevented sick leave days and prevented rehospitalisations will be calculated. A budget impact analysis will be conducted along with the economic evaluation to best inform the needs of decision makers regarding affordability and cost-effectiveness of the intervention.

### Measures taken to minimise/avoid bias

#### Randomisation and concealment of allocation

To control for known and unknown confounders and thus preclude selection bias, randomisation of patient-relative tandems will be conducted by means of a computer-based algorithm. Randomisation lists will adhere to a one-to-one study-group allocation ratio and will be stratified by centres with dynamically adaptable block sizes to prevent predictability and to ensure a balanced proportion of participants in both study conditions. To meet the challenges of a group intervention, randomisation blocks will include blocks with two, four, six, eight and ten patient-relative tandems. In each block size, the proportion of patient-relative tandems which will be allocated to the intervention or the control group is balanced. Once a study centre has recruited at least six patient-relative tandems, randomisation will be conducted with the appropriate block size. If a study centre has recruited two or four more patient-relative tandems before the first group session in the intervention group has taken place, they can be re-randomised and added to the PGIR and the control group. Randomisation lists will be centrally generated by the study statistician, who has no direct contact with clinicians or patient-relative tandems. To ensure allocation concealment, randomisation lists will be inaccessible to staff involved in the recruitment at the respective study centres. After recruitment of at least six patient-relative tandems (conducting the PGIR has to be feasible), study sites will report included patient-relative tandems to the leading study centre, which will report the allocation to the study sites as well as the patient-relative tandems. As the effect of the intervention was not moderated by any investigated variable in the study by Shimazu et al. [17], stratification for clinical characteristics will not be considered. However, central clinical variables (e.g., number of depressive episodes, duration of the current episode) will be gathered and their effects will be investigated in additional sensitivity analyses.

#### Documentation of recruitment

The recruitment of the study sample will be documented in detail in order to judge the representativeness of included patients compared to the basic population of inpatients in depression treatment in the included study sites. Basic socio-demographic data and specific reasons for refusal of study participation of all screened patients and relatives, as well as exclusions of screened patients due to the above-mentioned exclusion criteria, will be closely documented. Therefore, a standardised operating protocol regarding the process of recruitment and informing patients as well as relatives has been developed.

#### Blinding

Due to the nature of the intervention, blinding of therapists and patient-relative tandems is not feasible. The primary outcome will be analysed by the study statistician, who will be blinded to the allocation of patients.

#### Control of intervention adherence

The intervention will be manualised and all related materials (e.g., slides, handouts) are standardised and will be provided by the leading study centre. Additionally, therapists will be trained in conducting the intervention within an intensive workshop to ensure process quality and standardisation of the intervention. Training success will be verified by using simulated therapy situations in the style of the Objective Structured Clinical Examination (OSCE). Only staff reaching a previously defined standard will be authorised to conduct the intervention within this trial. To further ensure the integrity of the intervention, the leading study centre will provide supervision as well as peer consultation via telephone or video conference. Finally, group sessions will be audiotaped and rated using self-developed scales [71] to judge the adherence and competence of therapists (T_Int_, intervention, c.f. Table 1).

#### Control of treatment-related confounders

As the intervention is compared to TAU, and the treatment of the patient is not restricted in any way in either condition, different types of treatment received by the patients might influence the effects. Therefore, data on treatment received after discharge will be gathered on both the patient and the relative level (c.f. Table 1) and their effects will be investigated using sensitivity analyses.

### Statistical methods

#### Analyses

The primary outcome, i.e., estimated DFDs one year after discharge from inpatient depression treatment, will be analysed by means of an analysis of covariance (ANCOVA) (independent variables: intervention, study centre; dependent variable: DFDs; covariate: severity of depressive symptoms at baseline) comparing the number of DFDs one year after discharge from inpatient depression treatment between the intervention and the control group. The primary analysis will be performed according to the intention-to-treat principle including all randomised patients. Multiple imputations will be used to account for missing data. Analyses with an alternative method of dealing with missing data and using available data will only be performed as sensitivity analyses in order to test the robustness of the findings. Secondary interval-scaled outcomes will be tested using t-tests and analyses of covariance (ANCOVA, if baseline measurement of the outcome is available). Dichotomous outcomes will be analysed using Fisher’s exact test, chi-square test (depending on expected cell frequencies), or logistic regression (if covariates are present). To ensure the interpretability of the results, each inferential statistical analysis will be accompanied by descriptive analysis. Differences in time until rehospitalisation will be analysed using Cox regression. For parameters in which the variance cannot be calculated analytically (e.g., cost-effectiveness), bootstrap methods will be used. Longitudinal analyses will be performed using general (interval-scaled outcome) or generalised (binary or ordered categorical outcome) linear mixed models. For the primary confirmatory analysis, a result with a type-I error under 5 % (*p* < .05) will be considered as statistically significant. Every additional analysis is seen as exploratory. Accordingly, a correction of the type-I error inflation will not be applied.

#### Sample size calculation

The effect of the intervention in the study by Shimazu et al. [17] corresponds to an odds ratio of 0.09 (IG: 8 vs. CG: 50 % relapses), equivalent to a Cohen’s d of 1.33, which can be considered as a very large effect. However, the sample size was comparatively small, consisting of only 57 patient-relative tandems. Furthermore, there are some major differences to our study. First, the intervention in our study will be conducted by different therapists at several study sites. Second, our study is closer to routine conditions. Therefore, our study population will be more heterogeneous than in the study by Shimazu et al. [17]. A sample size of 128 patient-relative tandems one year after discharge (T_4_) is sufficient to detect a moderate effect of Cohen’s d = 0.50 (Cohen’s f = 0.25) with a power of 80 % by analysing the primary outcome via ANCOVA. In the study by Shimazu et al. [17], the dropout rate was about 5 %. As our study will be conducted under routine conditions, we estimate a conservative dropout rate of 10 % (*N* = 18) for the time of the intervention and a further 20 % lost to follow-up (*N* = 32). To reach a sample size of 128 patients under consideration of a total dropout rate of 30 %, a total of 180 patient-relative tandems shall be randomised.

## Discussion

Our study will provide information on the effects of PGIR as a maintenance treatment after inpatient depression treatment on central patient-related as well as relative-related outcomes, such as course of disease and social functioning in patients, as well as caregiver burden and expressed emotion in relatives. The PGIR approach evaluated within this trial faired very well in one small monocentre trial with a small sample size [17] but has never been evaluated within a multicentre trial. This is important, as small studies tend to overestimate effect sizes [21, 22]. The confirmation in a multisite study is therefore required. The brief intervention of our study is highly suitable for routine conditions regarding psychoeducation for relatives of patients in inpatient depression treatment [27]. The results of our study will be important, as they will close an evidence gap and can therefore clarify recommendations in current guidelines [5] and eventually foster the provision of PGIR as a sensible addition to depression treatment.

Potential limitations of our study include the fact that blinding of patients and relatives as well as therapists is not feasible. Furthermore, patients will differ widely regarding their clinical characteristics due to the broad inclusion criteria. Additionally, the group of relatives will be heterogeneous, as we are not focusing on a specific group of relatives, such as partners. Moreover, treatment and health care utilisation of patients and relatives will not be restricted. These aspects might dilute the effects of the intervention. However, this can also be seen as strength of our study, as the study population is likely to come close to routine conditions and hereby can ensure a high external validity of the results. A further limitation is that study participants have to self-administer a high number of measurement instruments. This might hinder the feasibility of the trial and reduce the response rate.

Relapse and rehospitalisations are major topics after inpatient depression treatment. PGIR is a highly promising approach to improve the course of disease in this burdensome disorder. However, high-quality studies dealing with PGIR as an additional intervention to stabilise outcomes after inpatient depression treatment are largely lacking.

## Trial status

Enrolment for the trial began in March 2015. At the time of manuscript submission, participants were still being recruited. Recruitment is expected to continue until March 2016. Data collection is expected to continue until March 2017.

## Abbreviations

ANCOVA: Analysis of covariance
CEA: Cost-effectiveness analysis
CSSRI-D: German version of the client sociodemographic and service receipt inventory
DEGS: German health interview and examination survey for adults
DFDs: Estimated number of depression-free-days
D-Lit: Depression literacy test
DRKS: German clinical trials register
FEF: Questionnaire on family atmosphere
FIMA: Questionnaire for health-related resource use
FQ: German version of the family questionnaire
ICD-10: International classification of disease, 10th revision
IEQ-EU: German version of the involvement evaluation questionnaire
IPQS-R: Scales for control attributions of the illness perception questionnaire for schizophrenia: relatives’ version
OSCE: Objective structured clinical examination
PCS: Perceived criticism scale
PHQ-9: 9-item version of the patient health questionnaire
PGIR: Psychoeducational group intervention for relatives
SCL-K-9: 9-item version of the symptom-ckecklist-90-R
SPSI-R-S: 25-item version of the social problem-solving inventory-revised
TAU: Treatment as usual
UTN: Universal trial number
WHODAS 2.0: WHO disability assessment schedule
